# Supplementary material for: Are carbon emissions trading and green financial instruments synergistic? -Comprehensive quantitative research based on content analysis
Source: PLoS One. 2024 Mar 7;19(3):e0298601. doi: 10.1371/journal.pone.0298601 (PMC10919601; doi:10.1371/journal.pone.0298601)
Supplement: S1 Data — (ZIP) [file pone.0298601.s001.zip › Data/地级市Z_F工作报告.zip!/╦─┤¿/├╝╔╜╩╨/└┤╫╘╣1⁄2╓┌║┼í╛┬φ┐╦╩2╛▌═°í┐_2018.docx]

尊敬的各位代表：

现在，我代表市人民政府，向大会报告工作，请予审议，并请市政协委员提出意见。

一、2017年工作回顾

过去一年，是我市发展进程中极为艰难、极不平凡的一年。面对结构调整、环保督察、转型提质等多重挑战，在省委省政府和市委坚强领导下，我们始终保持专注发展转型发展战略定力，坚持端正发展观念，坚定不移落实新发展理念，主动作为，求真做实，自我加压，攻坚克难，解决了一些多年想解决的难题，干成了一些力所能及、群众看得见摸得着感受得到的实事，推动了全市经济平稳健康发展、民生福祉持续改善、社会大局和谐稳定。全市地区生产总值1183.4亿元、增长5.3%，地方一般公共预算收入93.2亿元、增长6.8%，社会消费品零售总额486.9亿元、增长11.2%，城镇居民人均可支配收入31130元、增长8.5%，农村居民人均可支配收入15203元、增长9.1%，全社会固定资产投资完成1110.8亿元、下降4.5%，居民消费价格上涨1.6%，人口自然增长率4.1‰，城镇化率提高1.3个百分点。

一年来，我们主动推进产业转型，经济发展提质向好。现代农业蓬勃发展。第一产业增加值增长3.9%，居全省第五。新建高标准农田20.5万亩，粮食产量170.5万吨，粮食安全进一步加强。新建成农业标准化基地11万亩、现代农业产业融合示范园区15个，新培育农民专业合作社和家庭农场894个、“三品一标”农产品74个、地理标志证明商标2件，“丹棱桔橙”成为全省首个国家级农产品地理标志示范样板，“仁寿曹家梨”成为国家地理标志保护产品；中国泡菜城被农业部命名为首批国家现代农业产业园区，泡菜产业收入达到165亿元；“东坡味道”成为全省优秀农产品公用品牌，销售收入超过750亿元、增长12%。工业转型减量提质。针对工业中部分存在耗能高、污染重、安全隐患多的问题，有保有压，分类施策。低端落后产能有序退出，市内煤矿全部关停、压减煤炭产能130万吨，“地条钢”和落后粗钢全部取缔、压减钢铁产能93万吨。陶瓷产业全面实施“煤改气”，城市建成区10蒸吨及以下燃煤锅炉全部淘汰，二氧化硫排放量下降12.5%。分类处置“散乱污”工业企业，关闭380户、整治转型1136户、搬迁入园94户。经过一年的努力，工业质量效益明显提升，有效投资显著增强。全年新培育规上企业48户、四川名牌30个，工业品产销率达到98.1%，单位工业增加值综合能耗下降4%、水耗下降6%，规上工业企业资产负债率下降2.7个百分点，特别是支撑眉山工业的41户骨干企业产值、税收分别增长21%、28.7%。工业结构进一步优化，“4+4”主导产业占工业比重提升1个百分点、达86%，战略性新兴产业产值345亿元、增长15%，电子信息产业产值突破100亿元、增长30.6%。现代服务业量质齐升。第三产业增加值增长9.5%，占经济比重上升2.6个百分点。商贸物流繁荣发展，商品住宅销售面积增长11.3%，网络零售额增长28%，邮政业务量增长69.7%、居全省第一，电信业务总量增长30.2%，客货运输周转量增长7.5%。金融业快速发展，新增金融机构5家，全市存款增长16.1%、居全省第二，贷款增长15.7%、居全省第四。旅游经济发展强劲，市县（区）联动，成功举办首届樱花节、第八届东坡文化节暨首届四川音乐周、第九届中国泡菜博览会等重大会节，三苏祠景区接待人数增长55.7%，瓦屋山景区低山区展示性开放并被《中国国家地理》杂志评为“最美桌山”，柳江古镇荣获中国特色小镇，全市旅游接待总人数和总收入分别增长16.3%、20.9%。

一年来，我们主动争取试点示范，改革创新亮点纷呈。国省改革试点落户眉山。成功争取国家公交都市创建城市，为便民公交打下坚实基础。成为国家电信普遍服务补偿试点城市，有力助推“智慧眉山”建设。去年眉山还成为国家重大市政工程领域PPP创新工作重点市、全国殡葬综合改革试点市、全省农村养老服务体系建设提升水平型试点市、全省促进民营企业建立现代企业制度试点市等。洪雅县成为全省唯一国家森林康养基地试点县。眉山经验全国全省推广。促进返乡农民工创业工作获李克强总理肯定性批示，青神县土地集约节约工作获国务院办公厅通报表扬，矛盾纠纷多元化解机制改革经验得到最高人民法院推广，东坡区集体林权制度改革获全国先进，彭山区农村土地“四步流转、三方受益”改革经验全国交流，“东坡区中国泡菜城融合发展”“丹棱一县一品晚熟柑桔发展”入选全省十大农业供给侧结构性改革案例，东坡区、仁寿县创新现代农业开发助推乡村振兴被中央媒体报道。科技创新成果落地见效。实施市级以上科技项目100项，转化实施专利2000余件，新增省级科技企业孵化器3家、省级工程技术研究中心1家、高新技术企业9户，茂华食品成为全市首家国家技术创新示范企业，眉山经济开发区成功创建为省级高新技术产业园区，全市高新技术产业产值增长15.5%，科技对经济增长贡献率提升2个百分点、达到54%。

一年来，我们主动出击精准招商，开放合作取得突破。招商引资取得历史性突破。市县（区）携手联动，招到了一些过去想招而招不到、甚至想都不敢想的大项目好项目，引进百亿级、千亿级大项目8个，总投资3298亿元，其中404亿元的信利（仁寿）高端显示项目、1300亿元的恒大童世界和养生谷项目，分别为眉山建区设市以来最大的工业和服务业项目。全年引进投资10亿元以上重大履约项目27个，世界500强投资履约项目8个，战略性新兴产业项目23个，招商引资到位资金620亿元、增长7.9%。积极“走出去”推进海外招商，首次在国外设立眉山驻欧洲（伦敦）招商办事处。对外开放取得历史性突破。经国务院台办、国家发改委、商务部批准，海峡两岸产业合作区眉山产业园成功挂牌。高水平承办“瓦屋山杯”中国国际友好城市青少年足球邀请赛、第四届中日农业交流合作研讨会、中印关系国际研讨会等国际性活动，受到中外嘉宾广泛赞誉。32个国家地区、900余名外宾来眉考察，与法国盖亚克、泰国清迈、摩洛哥卡萨布兰卡等6个国外城市建立友好关系，眉山的吸引力越来越强，“朋友圈”越来越大，国际化的新眉山正豪迈起步！校地合作取得历史性突破。与香港大学、四川大学、北京外国语大学签订战略合作协议，与重点高校、科研院所、国家级实验室达成战略合作项目20个，引进高层次人才81名、外国专家15名。

一年来，我们主动抓好问题整改，生态环境明显改善。以前所未有的力度，推进中央、省环保督察反馈问题整改，中央环保督察组移交的293个举报受理件全部办结，一大批突出环境问题得到有效整治，全面关停瓦屋山自然保护区矿山和小水电站受到央视《新闻联播》肯定性报道。在全省率先成立公安环境犯罪侦查支队，立案32起，处理35人；特别是依法查处邓某非法加工煤焦油案，成为全省首例大气污染环境犯罪案件，有效震慑环境犯罪。强力实施“河长治水”。设立市县乡村四级河长1849名，关闭搬迁禁养区畜禽养殖场2217家、“种养循环”治理4992家，全市17条河流水质明显改善，岷江干流出境断面氨氮、总磷浓度分别下降33%和20.7%。强力实施“点长治气”。PM10、PM2.5浓度分别下降14.1%、19.5%，改善率分别居全省第四和第二；中心城区空气优良率达73.7%，优良天数同比基准年改善幅度居全省第一，受省通报表扬并获556万元奖励。强力实施“田长治土”。工业固废及危险废物综合处置利用率100%，化肥、农药使用量分别下降5.7%、4.9%，完成100个村生活垃圾无害化处理设施建设。持续推进“三大工程”建设。新造林6.7万亩，城乡绿化覆盖率提升2.7个百分点、达54.6%，新增和恢复水域面积26.3平方公里，新建成公园10个、幸福美丽新村162个、省级“四好村”100个。

一年来，我们主动解决民生难题，社会事业全面发展。脱贫攻坚决战决胜。19949名贫困人口、30个市定贫困村达标退出，3367户贫困群众易地搬迁，全市建档立卡10.94万人、316个贫困村整体达到脱贫退出标准，受到省委省政府表彰。社会保障更加有力。城乡低保标准、企业退休人员养老金全面提高，农村留守儿童和困境儿童关爱保护加强，改造棚户区10210套、农村危房637户，解决农村9.18万人安全饮水问题。新增城镇就业3.5万人，城镇登记失业率控制在4.14%。交通出行更加方便。千亿交通大会战成效明显，全年完成交通投资137亿元、增长20%；成都经济区环线高速眉山段建成通车；新增眉山成都动车2列次、达29列次，基本实现每小时一班；中心城区新增加城市公交线路3条、新投放城市公交车58辆、新建成公交候车亭56座；新（改）建农村公路481公里。教卫文体全面提升。新（改、扩）建校舍6.2万平方米、公办幼儿园21所，洪雅义务教育均衡发展通过国家督导评估，青神一名教师荣获全国十大“最美教师”称号。公立医院全部实行药品零差率销售，减轻群众就医负担1.2亿元，市人民医院建成三甲综合医院；在全省率先开展订单式培养医学专业学生100名，为基层卫生院储备人才。积极响应大众文化需求，开展“书香眉山·全民阅读”活动，文轩书城开业，市美术馆开馆。“江口沉银”水下考古、举办首届糖尿病50+论坛、承办全国特奥轮滑比赛、启明星龙舟队在中华龙舟大赛获奖，引发全国关注，提升眉山影响。社会保持和谐稳定。深入推进“平安眉山”建设，“5·24”特大跨境电信网络诈骗案等一批大案要案告破，刑事案件数下降27.2%，成为全国社会治安综合治理优秀市。生产安全事故起数、死亡人数分别下降24.8%、12.5%，未发生重特大安全生产事故。在全省率先成立地质灾害应急中心，地质灾害避险搬迁495户，连续九年零伤亡。12345服务热线受理群众诉求12.76万件，群众满意率94.5%。矛盾纠纷多元化解体系不断完善，信访总量保持全省末位。成功创建四川省文明城市，市民文明程度显著提高。民生承诺全部兑现。十大民生工程和20件民生大事全部完成，完成投入153.3亿元、增长14.5%，创历史新高，一大批群众关心的急难愁盼问题得到积极有效解决。眉山，正用民生温度，彰显共享发展新高度！其他各项事业全面发展。国家安全、国防动员、人民防空、民兵预备役等工作取得新进展，涉军群体权益保护得到加强，妇女儿童、产业工人、青少年、残疾人、红十字、慈善等事业加快发展，机关事务、哲学社会科学、档案、地方志、民族宗教、防震减灾等工作取得新成绩。

一年来，我们主动改进政务服务，发展环境不断优化。自觉接受市委领导，坚持市政府党组定期向市委报告工作。自觉接受市人大、市政协监督，落实市政府重大决策出台前向市人大常委会报告制度，定期向市政协常委会通报工作，认真办理人大代表建议115件、政协提案263件。加强审计和行政监督，大力整治“庸懒散浮拖”，公务员队伍纪律规矩意识明显增强，政府系统廉政建设和反腐败斗争不断深入，“三公”经费下降5%。行政效能进一步提升。全面推进“放管服”改革，减少审批前置条件276项，审批提速50%以上；取消村社证明事项84项，仅保留19项；深入推进“互联网+政务服务”，更多事项实现“信息多跑路、群众少跑腿”。政务服务进一步优化。创新建立“红色代办员”和每月“企业服务日”制度，帮助企业解决难题，减轻企业负担和成本27.2亿元，小微企业贷款净增54亿元、增长66.7%。深化商事制度改革，新登记个体工商户28411户、企业6052户，分别增长72.1%和19.4%。多项工作荣获全国先进。政务服务、法治财政建设、农业、城乡规划、疾控、未成年人思想道德建设、老龄、群众体育等工作被评为全国先进。

各位代表，困难中的转机来之不易，压力下的收获弥足珍贵。过去一年，经济社会发展取得新进步，这是在省委、省政府和市委坚强领导下，全市上下坚定以习近平新时代中国特色社会主义思想为指导，真抓实干、拼搏奋斗的结果！在此，我代表市人民政府，向在各个岗位辛勤工作的全市人民，向人大代表、政协委员、各民主党派、工商联、无党派人士、各人民团体、离退休老同志，向驻眉部队指战员、武警官兵、政法干警，向关心支持和参与眉山建设发展的社会各界人士，表示衷心的感谢并致以崇高的敬意！

在看到成绩的同时，我们也清醒认识到，全市经济社会发展中仍存在诸多困难和问题，主要是：市内区域发展不平衡，差距在加大；城镇化率仍低于全国全省平均水平，城镇化发展任重道远；农村基础设施薄弱，教育、文化、医疗等公共服务能力欠账较多，亟待提高；资源环境约束日益趋紧，污染防治任务艰巨；一些领域风险不容忽视；政府工作效率仍需提高，作风仍需改进。对此，我们一定直面问题、勇于改正、敢于担当、攻坚克难，绝不能辜负人民群众的期望！

二、2018年工作安排建议

今年是全面贯彻落实党的十九大精神的开局之年，是改革开放40周年，是市委确定的“落实年”。做好今年工作，意义十分重大。

当前，眉山发展正处于重大历史拐点，挑战前所未有，机遇也前所未有。为此，我们对形势必须要有清醒判断和理性认识。从挑战来看：一是底子薄、欠发达、不均衡的基本市情没有根本改变，特别是工业企业体量小、技术含量不高，千亿产业、百亿企业均在培育过程之中，加快发展、追赶发展任务艰巨。二是眉山地处四川盆地，静稳天气多，环保压力大，拼资源拼能耗的传统发展模式已不可持续，转型升级、绿色发展任务繁重。三是连续14年的高速增长，既打下基础，也带来压力，同时，国际国内经济形势复杂多变，不确定因素多发。这些困难也势必将在经济社会各个领域传导，给扩大就业、增加收入、维护稳定等民生事业发展带来新的挑战。从机遇来看：一是成都建设国家中心城市的战略，有利于眉山承接国家重大生产力布局。二是省委十一届二次全会提出的全面创新改革、天府新区建设、天府国际机场和国际空港新城建设、四川自贸区建设“四项重点工程”，均与眉山密切相关。三是贯彻中央环保督察要求，主动淘汰了一大批落后产能和“散乱污”企业，为发展腾出了空间。四是信利、恒大等一批百亿级、千亿级高端龙头产业项目将陆续开工建设，为新旧动能加速转换提供强大支撑。五是日益完善的现代立体交通、园区承载功能、城市基础设施配套，相对充足的人力资源，经过长期保护涵养形成的生态环境禀赋，为培育经济新动能提供了综合比较优势。

综合判断，机遇大于挑战，眉山正处于大有可为的历史机遇期。只要我们坚定以习近平新时代中国特色社会主义思想为指引，紧扣高质量发展要求，坚持稳中求进工作总基调，按照省委治蜀兴川“1234”战略谋划和市委治眉兴眉“135”总体布局，实事求是、真抓实干、久久为功，以钉钉子精神确保每一项工作、每一条措施都落到实处，我们就完全能应对各种风险挑战，完全能实现更有质量、更加实在、更可持续的发展，完全能开创繁荣富裕美好眉山新局面。

今年全市经济社会发展主要预期目标：地区生产总值增长7.5%，全社会固定资产投资增长10%，社会消费品零售总额增长10%，地方一般公共预算收入同口径增长7.5%，城乡居民人均可支配收入分别增长8%、9%，城镇登记失业率控制在4.3%以内，居民消费价格涨幅控制在3%以内，环境保护和节能减排完成省下达目标任务。重点抓好以下工作：

第一，着力深化改革创新，全面提升发展动能质效。改革创新是引领发展的第一动力。我们必须坚持以深化供给侧结构性改革为主线，加速释放改革新红利，激发高质量发展新动能。

深入推进“三去一降一补”。严格执行环保、质量、安全等法规标准，淘汰落后低端产能，妥善处置“僵尸企业”。加快建立多主体供给、多渠道保障、租购并举的住房制度，完善促进房地产市场平稳健康发展长效机制。推进产融结合，降低企业负债率。落实各项降低企业成本措施，继续执行阶段性降低企业社保缴费率政策。推进全国质量强市示范市创建，实施名牌发展战略，打造更多有影响力的品牌。

统筹推进重点领域改革。深化商事制度改革，大力推进“多证合一”“证照分离”，力争新登记个体工商户1.1万户、企业2100户以上。深化项目投资审批改革，推进并联、容缺审批和多评合一、多审合一、多图联审，加快推进26个省重点、157个市重点项目。深化投融资改革，积极探索“XOD+PPP”模式，继续壮大政府性投资基金。深化财税金融改革，建立政府性债务全口径监管机制，严格债务限额管理，稳妥有序推进互联网金融风险专项整治。统筹抓好环保、司法、综合行政执法等改革。

大力提升创新能力。深化与签约高校、科研院所合作，新建重点政产学研用联盟3个。强化以企业为主体的技术创新体系建设，实施重大科技成果转化项目30个以上，力争技术创新投资增长15%。抓好四川铂智汇、加州小镇、高端显示产业研究院等创新平台建设，启动眉山高新技术产业园区创建国家高新技术产业开发区，新增一批企业（工程）技术中心。新培育国家高新技术企业5户，全市高新技术产业产值增长12%以上。实施“眉山优才工程”，大力培养本土人才，引进一批战略科技人才和高水平创新团队，建设一批院士和博士后工作站。

第二，着力构建现代产业体系，全面提升产业发展质效。产业是推动高质量发展、建设现代经济强市的根本。我们必须紧抓产业不放，推动三次产业转型升级、融合发展，打造高质量发展新引擎。

加快建设都市现代农业强市。大力发展设施农业，加快推进“三园区五基地” 建设。加快中法农业科技园、泰国正大生态养殖全产业链、中华枇杷园等39个现代农业项目建设，完成投资85亿元以上。大力推进晚熟柑桔 “三百工程”建设，办好首届晚熟柑桔节，在眉山泡菜之外再造一个全国有影响力的眉山晚熟柑桔品牌。实施“东坡味道香飘世界行动”，办好第16届中国食品安全年会、中国国际泡菜食品博览会。新培育“三品一标”农产品10个、新型农业经营主体300个以上。坚守耕地红线，落实粮食安全责任制，保持粮食生产稳定。

大力发展先进制造业。深入实施“眉山制造2025”，重点发展电子信息等“3135”产业，突出抓好90个投资亿元以上工业及技改项目，加快推进信利、千禾味业、万华化学、阿格瑞新材料、百途医药等项目建设，完成工业投资310亿元，力争规上工业增加值增长8%、战略性新兴产业产值增长15%。深化“互联网+先进制造业”，建成华为大数据中心，打造四川特色大数据产业外包服务基地。实施“1258”企业培育计划，支持金象赛瑞、启明星铝业、中车眉山公司等41家骨干企业做大做强。实施“小升规、规转股、股上市”行动，新增规上工业企业50户，60户民营企业建立现代企业制度，新增挂牌、上市企业20户。

加快发展现代服务业。实施服务业“115”工程，优先发展六大先导型服务业，重点抓好97个投资千万元以上项目，第三产业增加值增长9%以上。加快发展高端文旅产业，实施旅游三年倍增计划，加快恒大童世界、中铁生态城、七里坪、丹棱国家乡村公园、青神竹编产业园等建设，推进瓦屋山、黑龙滩、三苏祠创5A级旅游景区，瓦屋山景区今年5月全面开山。大力发展金融、现代物流、节能环保服务等生产性服务业，人民币存贷款余额分别增长8%和9%。提质发展家政、商贸等生活性服务业。积极培育健康养老、文化体育等消费热点，创建“放心舒心消费城市”。

第三，着力扩大开放合作，全面提升招商引资质效。开放是眉山发展的必由之路。我们必须瞄准国际、对标先进，大力实施“开放升级”行动，推进更大范围、更宽领域、更高层次开放合作。

强化精准精品招商。围绕优势主导产业，强化“亩产论英雄”综合评价，突出招强引优、以商引商、产业链招商，集中优势资源招引大项目好项目，力争引进“三类500强”或投资10亿元以上项目20个，到位资金660亿元。加快新项目落户建设，重点抓实10亿元以上重大优质项目落地，提高履约率、开工率、达产率。

深化内外交流合作。积极参与“一带一路”建设，密切与港澳地区、国外友城、海外华侨华人及其社团的交往合作，高水平办好联合国教科文组织2018“历史村落的未来”国际会议、东坡文化国际学术高峰论坛、国际竹产业交易博览会、国际长板速降公开赛等国际活动。高水平规划建设海峡两岸产业合作区，加强对台招商推介，促进一批优质台资企业落户眉山。深化区域合作，加快推进与成都、甘孜等市州合作项目落地落实。

提速建设天府新区。积极参与天府新区总规修编，完成环天府新区经济带规划。坚持产城一体，完善交通能源、公共服务、生态绿地等城市功能，加快格力电工、中建新材料产业园等一批重点项目建设，引进落实一批具有带动性、引领性的重大新兴产业项目，培育高端化产业集群，提高投资强度和产出效益，确保发展速度、质效高于全市平均水平。

第四，着力新型城镇化建设，全面提升城乡统筹质效。区域协调、城乡融合是高质量发展的重要内容。我们必须坚持繁荣城市与振兴乡村并举，加快构建新型城乡形态，实现城乡协调发展。

大力推进城镇繁荣发展。加快城市总规修编，推进中心城区绿地、足球和篮球场、“城市家具”等专项规划，完成历史文化名城保护规划。继续实施城镇基础设施千亿大会战，加快海绵城市、智慧城市、公交都市建设，力争完成中心城区“五纵六横”道路黑化工程。实施“教体医卫腾飞行动”，引进一批知名机构来眉办学办医，加快北外附属东坡外国语学校、川大眉山校区、四川药科职业学院、华西第二医院眉山市妇女儿童医院、尤文图斯足球学院培训中心建设。实施“宜居县城建设行动”，深化“百镇建设行动”。加强城市管理，提高精细化智能化水平。户籍人口城镇化率提高1.4个百分点。

加快推进美丽乡村建设。实施乡村振兴“十大行动”，建成幸福美丽新村137个、市级“四好村”110个。全面深化农村改革，建立农村土地增值收益全部用于脱贫攻坚和乡村振兴的政策，支持市属国有企业参与乡村振兴规划建设。加强农村水电气路讯等基础设施建设，巩固提升农村安全饮水，新（改）建“四好农村路”400公里。改善农村住房条件，完成土坯房改造8447户。推进农村“厕所革命”，新（改）建卫生厕所119个。开展农村垃圾分类百村示范行动，无害化处理率80%以上。加强传统村落保护利用，争创一批国省级历史文化名镇名村和传统村落。加强农村公共文化建设，办好农民夜校，培育文明乡风、良好家风、淳朴民风。

构建现代基础设施网络。继续实施千亿交通大会战，加快推进“8轨16路2机场1航道”建设，力争完成投资160亿元。实施全域安全饮水工程，加快推进眉彭、眉青供水管道和丹棱青衣江引水工程等项目，建成投运市三水厂。完善新一代信息网络基础设施，实施“智慧眉山”行动，推进“一中心、三平台、多系统”建设。

第五，着力加快生态文明建设，全面提升绿色发展质效。生态环境是眉山最大的财富。我们必须坚持“绿水青山就是金山银山”，做好生态建设和环境治理“加减法”，着力为群众提供更优质的生态产品。

强力推进污染防治。坚决打好大气、水、土壤污染防治三大战役。坚持铁腕治霾，抓好“减排、压煤、抑尘、控秸、治车”，确保优良天数率达70%以上，让蓝天白云成为“常客”。纵深推进河（湖）长制，加快毛河、醴泉河、思蒙河、球溪河、金牛河等重点小流域治理，新（改）建污水处理厂（站）205座，完成通惠河城区段截污工程。推进县级饮用水水源地整治，加强农业面源污染治理。开展土壤环境状况调查，推进生活垃圾强制分类试点，完成医疗废物集中处置工程建设。继续抓好中央和省环保督察反馈问题整改，决不允许反弹回潮，让群众真真切切感受到环境越来越好！

加强生态建设保护。继续推进“三大工程”建设，新造林6万亩，恢复和新增水域面积20平方公里，新建和续建公园25个，加快创建国家森林城市。大力推进资源节约集约和循环利用，积极开展绿色社区、绿色出行等行动。健全企业排污许可、环境信用评价、信息强制性披露等制度，强化监管执法，坚决制止和惩处破坏生态环境行为。推进领导干部自然资源资产离任审计，实行生态环境损害责任终身追究。完成水土保持政府目标责任制考核。

第六，着力增进民生福祉，全面提升社会事业质效。人民对美好生活的向往就是我们的奋斗目标。我们必须持之以恒保障和改善民生，让发展更有“温度”、幸福更有“质感”。

抓好十大民生工程。坚持民生投入和为老百姓办实事只增不减，全部新增财力和八成以上财政性支出用于民生。围绕老百姓最盼、最急、最忧、最怨的问题，继续实施十大民生工程和20件民生大事，完成投入155亿元以上。

办好优质公平教育。深化教育改革，统筹各级各类教育协调发展，加强教师队伍建设，提升教学质量和教育水平。加强“全面改薄”和中心城区学校扩容工作，新（改、扩）建校舍5万平方米，加快齐通路幼儿园、东坡岛小学、苏辙中学等12个项目建设，着力缓解中小学生择校热、大班额等问题。

积极抓好就业创业。完善公共就业创业服务体系，加快市公共实训基地、市人力资源市场建设，优化就业创业环境。加大高校毕业生、“散乱污”企业失业员工等群体的就业帮扶，大规模开展职业技能培训，多渠道开发就业岗位，新增城镇就业2.6万人以上。

加快建设健康眉山。深化医药卫生体制改革，坚持“三医联动”，推进医联体、专科联盟创建，加快市人民医院二期建设、市中医医院迁建、市疾控中心和县级医院扩建，积极缓解群众“看病贵、看病难、看病烦”问题。全面启动国家卫生城市创建。做好新形势下计划生育工作。抓好农村养老服务体系建设试点，推广“1234”农村养老服务模式，加快市老年大学建设。开展“双安双创”，建好食品药品智慧监管平台。广泛开展全民健身活动。

大力繁荣文化事业。加快国家历史文化名城创建，推进文化传承创新“七个一”工程，重点打造三苏祠精品博物馆，规划建设江口沉银博物馆和遗址公园。深化文化体制改革，完善公共文化服务体系，加快“五馆一中心”、当代艺术中心、市档案中心建设，市电视台综合频道建成高清频道，广泛开展文化惠民活动，为广大群众提供丰富的精神食粮。启动全国文明城市创建，大力弘扬和践行社会主义核心价值观。

巩固提升脱贫成效。聚焦“两不愁、三保障”“四个好”，完成1.1万人脱贫和3785人易地扶贫搬迁任务。建立脱贫攻坚巩固提升长效机制，抓好 “回头看”“回头帮”。落实教育、医疗、住房等保障措施，改善城市困难家庭生活条件。继续做好阿坝州金川县、茂县对口帮扶。

加强创新社会治理。深化“平安眉山”建设，健全城市安全体系，强化社会治安防控体系建设，大力开展扫黑除恶专项斗争，加快吸毒人员监管场所、青少年毒品预防教育基地等建设。严格落实安全生产责任制，开展重点行业重点领域安全生产大整治，坚决防范重特大事故。完善社会治理体系，抓好“七五普法”“法律七进”，强化网格化管理服务和社会稳定风险评估，加强“两法衔接”，维护社会和谐稳定。

统筹发展各项事业。加强国防动员体系建设。加强应急管理，抓好防汛抗旱、极端天气应对和地质灾害防治。健全农村留守儿童、妇女关爱服务体系。统筹抓好民族宗教、信访、地方志、保密、档案、气象、防震减灾、供销、贸促、粮食、红十字、慈善、残疾人、关心下一代等工作。

三、建设“实干、高效、廉洁”的人民满意政府

中国特色社会主义已经进入新时代，新时代要有新气象，新时代要有新作为。我们要以习近平新时代中国特色社会主义思想统领各方面工作，树牢“四个意识”，坚定“四个自信”，旗帜鲜明讲政治，全面加强政府系统党的建设，坚决维护以习近平同志为核心的党中央权威和集中统一领导，坚决落实党中央国务院、省委省政府和市委各项决策部署，全力建设让党放心、人民满意的政府。

加强法治政府建设。全面落实《法治政府建设实施纲要》，不断提高运用法治思维和法治方式推动工作、服务群众的能力。坚持依法决策，全面推进政府法律顾问制度，落实重大行政决策公众参与、专家论证、风险评估、合法性审查、集体讨论决策等制度。自觉接受市人大及其常委会的法律监督、工作监督和政协的民主监督，坚持市政府党组定期向市委报告工作和市政府重大决策出台前向市人大常委会报告制度，认真办理人大代表建议和政协提案。主动听取民主党派和社会各界的意见，主动接受社会和舆论监督，强化审计监督，支持监察监督、司法监督。严格落实行政执法责任制，规范公正文明执法。完善统计体制，做好第四次全国经济普查工作。

加强服务型政府建设。加强学习培训和调查研究，不断掌握新知识、熟悉新领域、开拓新视野，努力增强“八种本领”，用更专业、更有效的办法解决发展中的问题，把高质量要求体现到政府工作方方面面，做好新时代的答卷人。深化“放管服”改革，继续清理取消不合法不合理证明和手续，着力解决办证难、办事难问题；深入推进“互联网+政务服务”，加快实现“一号申请、一窗受理、一网通办”，努力让企业和群众办事像“网购”一样方便。以简政减税减费为重点，进一步优化营商环境，建立营商环境评价机制；强化事中事后监管，营造公平竞争发展环境。政府要带头讲诚信，依法履行合同约定，决不搞朝令夕改，决不搞新官不理旧账！

加强廉洁政府建设。落实全面从严治党主体责任，扎实开展“不忘初心、牢记使命”主题教育。落实中央八项规定及其实施细则和省委、市委贯彻落实办法，坚决反对特权、不搞特权，驰而不息纠正“四风”。加强重点领域制度建设，盯紧公共资金、国资国企、公共资源交易等领域廉政风险点，管住关键环节。深入推进政务公开，及时主动回应社会关切，走好新形势下的群众路线，做到群众合理诉求“马上办”。健全正向激励和容错纠错机制，大力整治“庸懒散浮拖”行为，严肃查处群众身边的腐败问题，树立勤政廉洁务实高效的良好政风。

各位代表！征程万里风正劲，重任千钧再扬鞭。让我们紧密团结在以习近平同志为核心的党中央周围，在市委的坚强领导下，始终与全市人民站在一起、想在一起、干在一起，以永不懈怠的精神状态，和一往无前的奋斗姿态，共同书写建设繁荣富裕美好眉山的时代答卷！
